# Increased carotid intima-media thickness in Brazilian adolescents with type 1 diabetes mellitus

**DOI:** 10.1186/s13098-016-0190-0

**Published:** 2016-11-11

**Authors:** Maria Fernanda Gontijo Sepulveda Fusaro, Jovita Lane Soares Santos Zanini, Ivani Novato Silva

**Affiliations:** 1 Divisão de Endocrinologia Infantil e do Adolescente-Departamento de Pediatria, Faculdade de Medicina/Hospital das Clínicas, Universidade Federal de Minas Gerais, Av Alfredo Balena 190, s/267, Belo Horizonte, MG 30130-100 Brazil; 2 Department of Anatomy and Image, Hospital das Clínicas, Medical School, Universidade Federal de Minas Gerais, Belo Horizonte, Brazil

**Keywords:** Diabetes Mellitus, Carotid intima-media thickness, Atherosclerosis, Risk Factors, Ultrasonography

## Abstract

**Background:**

Increased carotid intima-media thickness (CIMT), a marker of subclinical atherosclerosis, is an independent predictor of future cardiovascular events, and has been reported in children with various chronic diseases, including type 1 diabetes mellitus (DM1).

**Objectives:**

Evaluate CIMT and its association with cardiovascular risk factors in Brazilian adolescents with DM1.

**Methods:**

Cross-sectional study of 118 adolescents, 57 with DM1 and no chronic complications related to the disease, and 61 healthy individuals. Clinical, biochemical, and high-resolution B-mode ultrasonographic evaluations according to the Consensus Statement of the American Society of Echocardiography CIMT Task Force were performed.

**Results:**

Adolescents with diabetes (66.6% female) were 14.5 ± 2.9 years old and had 9.0 ± 4.0 years of disease duration. The healthy adolescents (62.3% female) were 14.3 ± 2.6 years old. All the adolescents had blood pressure within their reference ranges. In 66% of DM1 adolescents the systolic blood pressure was >50th percentile. Increased CIMT was observed in adolescents with diabetes compared with those in the control group: 0.53 vs 0.51 mm (p < 0.004) on the right side, and 0.55 vs 0.51 mm (p < 0.001) on the left side. CIMT presented independent and positive associations with diabetes duration, total cholesterol level, low-density lipoprotein cholesterol level, and systolic blood pressure percentile in DM1 adolescents.

**Conclusions:**

Increased CIMT was observed in young Brazilian adolescents with DM1, and was associated with cardiovascular risk factors. CIMT assessment may be useful for the early identification and monitoring of cardiovascular risk in this age group.

## Background

Diabetes mellitus is associated with a high risk of cardiovascular disease (CVD). Carotid intima-media thickness (CIMT) is a noninvasive index of subclinical atherosclerosis and is significantly increased in type 1 Diabetes Mellitus (DM1) patients as compared to control subjects [1]. Long-term diabetes-related complications account for most of the increased morbidity and mortality associated with the disease, and longitudinal studies indicated that atherosclerotic processes in the endothelium begin in childhood and progress rapidly in the presence of risk factors [2]. Therefore, special attention should be given to monitoring and controlling risk factors in children with diabetes because of their high risk of CVD in the future [3]. Carotid ultrasound has been used in the evaluation of intima-media thickness (IMT) due to its accessibility and low cost. Increased CIMT correlates with cardiovascular outcomes such as increased risk of stroke and coronary artery diseases [4]. Significant thickening of the endothelial wall has also been reported in children with various chronic diseases, including DM1 [5, 6]. Factors such as age at DM1 onset, poor glycemic control, high levels of low-density lipoprotein cholesterol (LDL-C) and systolic blood pressure (SBP) have been associated with increased CIMT in pediatric patients [7]. However, the relationship between changes in intima-media thickness, and physiological or pathological conditions in childhood has not been fully elucidated. Moreover, as far as we know, no Brazilian data has been published on CIMT in DM1 adolescents. Therefore, this study aimed to evaluate CIMT and its relationship with cardiovascular risk factors in Brazilian adolescents with DM1 followed-up at a single university center.

## Subjects and methods

This cross-sectional study was approved by the Institution Research Ethics Committee and the adolescents were included after agreeing to participate.

One hundred and eighteen adolescents were evaluated: 57 DM1 patients and 61 healthy individuals.

From an universe of 320 potentially eligible patients with DM1, 57 adolescents 10–20 years old who had a disease duration >5 years, and agreed to participate, were included in the study. They were diagnosed according the American Diabetes Association criteria [8], and had been in regular follow-up at the Pediatric Endocrinology Service of the University Hospital, under routine clinical and laboratory evaluation. None of them had history of chronic, autoimmune diseases or regular use of medication, but the diabetes. They had been under basal-bolus, intensive insulin regimen (most receiving NPH-ultra rapid insulin) of multiple daily doses. They had neither acute diabetic complications nor chronic diabetes-related macro or micro-vascular complications, such as diabetic neuropathy, retinopathy or nephropathy, as assessed by clinical evaluation, fundus examination or microalbuminuria detection, respectively.

The healthy adolescents were recruited from a public school, and matched to the DM1 group for age and gender. The inclusion criteria consisted of being in good health. The exclusion criteria were the presence or a history of any chronic disease, and continuous use of medication.

All participants underwent a clinical and biochemical evaluation. A complete physical examination was conducted by the same researcher. The anthropometric parameters assessed according to the World Health Organization (WHO) criteria were measured using portable digital scales (G-Tech^®^) to the nearest 0.1 kg for weight, and a wall-mounted stadiometer to the nearest 0.1 cm for height [9]. The body mass index (BMI) Z-scores were classified according to the WHO established criteria [9]. Pubertal staging for females breast development and for testes size/genitalia for males were evaluated using the Tanner criteria [10].

Blood pressure was measured with the subjects in the supine position, using a Tycos sphygmomanometer, and appropriate cuffs for each participant. Blood pressure was analyzed following the fourth report on the diagnosis, evaluation, and treatment of high blood pressure in children and adolescents, according to age and height [11] and according to the percentile distribution [12].

Following overnight fasting, blood samples were collected between 7 and 8 a.m. for the biochemical assessment. Serum total cholesterol (Total-C) and fractions, and triglycerides were measured by colorimetric methods (VITROS® 5.1 FS Chemistry System, Buckinghamshire, UK), and analyzed according to the I Guidelines for prevention of atherosclerosis in childhood and adolescence [13]. The mean of the last three serum glycated hemoglobin (HBA1c) values for each patient was obtained in the last year of follow-up. They were assessed (immunoturbidimetric assay) and analyzed according to the recommendations of the American Diabetes Association [14].

### Assessment of carotid artery intima media thickness (CIMT)

High-resolution B-mode ultrasonography was performed by a single experienced specialist in radiology and diagnostic imaging to measure the intima-media layer thickness and evaluate the colour Doppler flow characteristics of the carotid arteries. The recommendations of the Consensus Statement from the American Society of Echocardiography Carotid Intima-Media Thickness Task Force were followed [15].

The exams were conducted using a Philips device model HD11XE, with a high-resolution multi-frequency linear transducer adjusted to 12 MHz. B-mode colour photographic and Doppler velocimetry documentation was performed using image-acquisition software Image Explorer 3.1 for Windows.

After resting for 10 min, the subjects were evaluated in the supine position with the neck slightly extended and inclined to 45°. Longitudinal and transverse sections of the right and left common carotid arteries (CCA), carotid bulbs, internal carotid arteries, and external carotid arteries were obtained using B-mode and colour Doppler ultrasound.

The ultrasound evaluation focused on the identification of the intima-media layer and distinction of focal atherosclerotic plaques. Three measurements of the IMT of each right and left common carotid arteries were obtained in the 15 mm stretch below the bulb region, free from focal plaques, where the double lines pattern of the IMT can be clearly observed. Multiple measurements were averaged to report a mean IMT value for each artery. Colour Doppler flow examination of the internal and external common carotid arteries was conducted using technical parameters specific to the carotid arteries. Pulsed Doppler ultrasound was performed in the most distal segments of the internal and external common carotid arteries accessible to the exploratory probe with an insonation angle less than or equal to 60° to obtain quantitative information through spectral analysis. The equipment settings used were the same as the ones used for the qualitative analysis.

### Statistical analysis

For the analyses, the R version 2.15.0 and EpiInfo version 6.04 software were used, in the public domain. Categorical and quantitative variables were described as frequencies, percentages, measures of central tendency (mean and median), and dispersion (standard deviation). Comparisons between the response variables and binary covariates were performed by using the Student *t* test, when the usual model assumptions (normality and homoscedasticity) were met. Comparisons of variables with more than two categories were performed by using the *F* test (analysis of variance), as the assumption of normality was also satisfied. Normal distribution of the variables was tested using the Shapiro–Wilk test for normality and Levene test for homoscedasticity (homogeneity among the variances).

The Pearson correlation coefficients were calculated to compare outcome variables and quantitative covariates. The mean CIMT measurements were compared between the diabetic and control groups by using the Student *t* test.

A p < 0.05 was considered as statistically significant.

For the multivariate analysis, linear regression models were developed to explain each response variable. In the first step of fitting the regression model, all covariates with p ≤ 0.25 in the univariate analysis were included. The variables were then removed in a step-by-step process up to the point the final model included only those with statistical significance (p ≤ 0.05) and clinical importance. The fitting was adjusted from the residue analysis.

## Results

The 57 DM1 adolescents (66.6% female) were 14.5 ± 2.9 years old and had disease duration of 9.0 ± 4.0 years. Most of them (95%) had already started puberty and had normal weight for height (96.5%); only two patients were underweight according to the BMI percentile for age. Adolescents with DM1 had both SBP and diastolic blood pressure (DBP) within the normal range for age and height; 66% of them presented SBP >50th percentile.

The clinical and biochemical characteristics of the adolescents are presented in Table 1.

**Table 1:** Clinical and Biochemical (mean ± SD) characteristics of DM1 adolescents and healthy controls

Characteristics	Adolescent population		p value
	57 DM1 patients	61 healthy controls	
Age (years)	14.5 ± 2.9	14.3 ± 2.6	1.0
Gender	F 38 M 19	F 30 M 23	0.374
Puberty (Tanner)^a^			
Initial	29	31	1.0
Advanced	28	30	
Z BMI	−0.40 ± 0.9 (20.0 ± 2.4)	−0.40 ± 0.9 (20.8 ± 2.2)	1.0
SBP	103.1 ± 10.2	110.1 ± 8.5	<0.001
DBP	66 ± 10.4	72 ± 7.2	<0.001
Triglycerides (mg/dL)	88.5 ± 46.5	72 ± 26.8	0.022
Total-C (mg/dL)	178.3 ± 31.9	148.5 ± 19.1	<0.001
LDL-C (mg/dL)	97.0 ± 24.3	84.7 ± 17.8	0.002
HDL-C (mg/dL)	61.4 ± 17.3	49.6 ± 8.4	<0.001
HbA1c	9.7 ± 2.0	NA	
Duration of diabetes (m)	109.0 ± 35.6	NA	

*SBP* systolic blood pressure, *DBP* diastolic blood pressure (mm/Hg), *NA* not applicable

^a^ *Tanner 2/3* initial puberty; *Tanner 4/5* advanced puberty

Most of DM1 adolescents (75%) had poor glycemic control. Only 35% showed Total-C levels within the reference range, and 66% showed triglyceride levels within the reference range.

The measurements of the right and left CIMT of the adolescents with DM1 are presented in Table 2. An increased CIMT on both sides was observed in adolescents with DM1 as compared with the adolescents in the control group.

**Table 2:** Increased RCIMT and LCIMT values in 57 DM1 adolescents compared with 61 healthy controls

Characteristics	n	Mean ± SD	Min	1Q	Median	3Q	Max	p value^a^
Right side								
Patients	57	0.53 ± 0.05	0.45	0.51	0.53	0.58	0.66	*0.004*
Control group	61	0.51 ± 0.05	0.41	0.48	0.51	0.56	0.53	
Left side								
Patients	57	0.55 ± 0.04	0.47	0.51	0.54	0.58	0.64	<*0.001*
Control group	61	0.51 ± 0.04	0.44	0.48	0.51	0.55	0.61	

*RCIMT* right carotid intima media thickness, *LCIMT* left carotid intima media thickness

^a^Student *t* test

CIMT presented independent and positive associations with Total-C level, low-density lipoprotein cholesterol level (LDL-C), diabetes duration and SBP percentile in DM1 adolescents.

The Total-C and LDL-C concentrations showed moderate and significant positive correlation with CIMT both on the right (*r* = 0.397, p < 0.002 and *r* = 0.405, p < 0.002 for Total-C and LDL-C, respectively) and left sides (*r* = 0.423, p < 0.001 and *r* = 0.463, p < 0.001, respectively).

High Total-C and LDL-C levels were significantly associated with increased CIMT. CIMT measurements were lower in the patients presenting Total-C within the reference range, when compared to the patients showing high levels and, also to those presenting borderline values. The adolescents with DM1 with high Total-C levels showed CIMT = 0.57 ± 0.04 mm on the right side, whereas those with borderline values showed CIMT = 0.53 ± 0.05 mm, and those with Total-C within the reference range presented CIMT = 0.52 ± 0.04 mm (p < 0.012). The same association was observed on the left side.

The same trend was observed in the adolescents with DM1 with high LDL-C levels showing, on the left side, increased CIMT (0.59 ± 0.03 mm) associated to high LDL-C levels, whereas those patients with borderline values showed CIMT = 0.56 ± 0.04 mm, and those with values within the reference range CIMT = 0.54 ± 0.04 mm (p < 0.015). On the right side, the adolescents presented CIMT = 0.57 ± 0.03 mm, whereas those with borderline values showed CIMT = 0.55 ± 0.06 mm. Those with values within the reference range showed CIMT = 0.52 ± 0.04 mm, not reaching statistical significance (p < 0.119).

Diabetes duration showed a moderate and significant positive correlation with CIMT both on the right (*r* = 0.414, p < 0.001) and left sides (*r* = 0.461, p < 0.001).

We didn’t find a significant association between CIMT and BP data both on the rigth (SBP: *r* = 0.153, p = 0.261, and DBP: *r* = 0.168, p = 0.214) and left sides (SBP: *r* = 0.243, p = 0.072, and DBP: *r* = 0.195, p = 0.150).

The SBP percentile was strongly correlated with CIMT on both sides (*r* = 0.768, p < 0.001 on the right side and *r* = 0.590, p < 0.001 on the left side). Moreover the adolescents with SBP >50th percentile showed significantly higher CIMT measurements than the patients with SBP ≤50th percentile in both the right (0.58 × 0.51 mm, p < 0.001) and left sides (0.58 × 0.53 mm, p < 0.001).

We found no association between the HBA1c and CIMT values.

A poor correlation was found between the BMI and CIMT values (*r* = 0.337, p < 0.010) and the triglyceride levels and CIMT values (*r* = 0.278, p < 0.036).

In the multivariate analysis DM1 adolescents with Total-C within the reference range showed, on average, a 0.03-unit less (95% CI 0.001–0.01) in CIMT measurements on both sides, when compared with the patients with high cholesterol levels. CIMT measurements were not different when comparing patients with high Total-C levels and those presenting borderline values (p = 0.118 on the right side and p = 0.089 on the left side).

The assessed CIMT increased by 0.006 unit on average (95% CI 0.001–0.01) on both the left and right sides, with each 10-unit increase in LDL-C concentration. On the left side, patients with LDL-C within the reference range had, on average, 0.04-unit less in CIMT as compared with the patients with high LDL-C values. CIMT measurements were not different when comparing patients with high LDL-C levels and those presenting borderline values (p = 0.313).

A positive association was found between CIMT and diabetes duration: at every 10 months of the disease, the mean CIMT evaluated on both sides showed an average increase of 0.004 unit (95% CI 0.001–0.007).

The adolescents with DM1 with SBP >50th percentile had, on average, a 0.06-unit increase in CIMT on the right, and a 0.05-unit increase on the left sides as compared with the adolescents with SBP ≤50th percentile (p < 0.001). The results of the multivariate analysis for the right and left carotid arteries are summarized in Tables 3 and 4, respectively.

**Table 3:** Association between the risk variables and RCIMT in 57 DM1 adolescents in the multivariate analysis

Variables	RCIMT			
	I	II	III	IV
Categorical				
Total C				
Borderline	−0.02 (−0.05 to 0.01)	–	–	–
Normal	−*0.03 (*−*0.06 to* −*0.003)*	–	–	–
LDL-C				
Borderline	–	–	–	–
Normal	–	–	–	–
SBP percentile	–	–	–	*0.06 (0.05*–*0.08)*
Quantitative				
DM1 duration	*0.0004 (0.0001–0.0007)*	*0.0004 (0.0001–0.0007)*	*0.0004 (0.0001–0.0007)*	*0.0003 (0.0001–0.0005)*
Total C	*–*	*0.0004 (0.0001–0.0008)*	*–*	*–*
LDL-C	–	–	*0.0006 (0.0001–0.001)*	–

OR (confidence interval)

*RCIMT* right carotid intima media thickness

– not present in the model

**Table 4:** Association between the risk variables and LCIMT in 57 DM1 adolescents in the multivariate analysis

Variables	LCIMT				
	I	II	III	IV	V
Categorical					
Total C					
Borderline	−0.02 (−0.05 to 0.003)	–	–	–	–
Normal	−*0.03 (*−*0.05 to* −*0.003)*	–	–	–	–
LDL-C					
Borderline	–	–	−0.02 (−0.06 to 0.02)	–	–
Normal	–	–	−*0.04 (*−*0.08 to* −*0.01)*	–	–
SBP percentile	–	–		–	*0.05 (0.03*–*0.06)*
Quantitative					
DM1 duration	*0.0004 (0.0001*–*0.0007)*	*0.0004 (0.0001*–*0.0007)*	*0.0005 (0.0002*–*0.0008)*	*0.0004 (0.0001*–*0.0007)*	*0.0004 (0.0001*–*0.0006)*
Total C	–	*0.0004 (0.0001*–*0.0007)*	–	–	–
LDL-C	–	–	–	*0.0006 (0.0002*–*0.001)*	–

OR (confidence interval)

*LCIMT* left carotid intima media thickness

– not present in the model

## Discussion

In the present group of DM1 adolescents it was shown an increased CIMT when compared with the healthy controls of the same age, through a systematic assessment by the same examiner, by using the same equipment and technique. To our knowledge, this is the first study to investigate the values of CMIT in Brazilian adolescents with DM1.We also observed that disease duration, higher concentrations of Total-C and LDL-C, and SBP >50th percentile were associated to the thickening of the carotid intima-media layer.

CVD remains as the leading cause of mortality in patients with diabetes in adulthood. The thickness of the intima-media layer of the carotid arteries is a marker of subclinical atherosclerosis and has been widely used to assess vascular health, including that of children with diabetes [6]. Increased CIMT in DM1 children has been related to early atherosclerosis changes [16, 17], and also been associated with diabetic microvascular complications as nephropathy and retinopathy [18, 19]. Abnormalities in myocardial function were recently reported, besides early vascular endothelial dysfunction induced by DM1. It was also reported that early atherosclerotic changes in the adolescents were associated with diabetic nephropathy, supporting that diabetic microangiopathy and macroangiopathy are related to each other in type 1 diabetic patients [20].

Although evaluation of CIMT as an early marker of atherosclerotic changes of the arterial wall has been recognized for >20 years in young DM1 patients [21] it has not been recommended in clinical practice. New possibilities to predict future cardiovascular events and endothelial dysfunction in DM1 children had already been reported but are not recommended [22].

It should be taken into consideration that an age- and puberty progression-related slight increase in the thickness of the intima-media layer is reported in studies with healthy children and partly represents an adaptative response to physiological increases in blood volume and pressure [23, 24]. The lack of standardization of CIMT values in this age group represents a hindrance to the wider use of CIMT measurements in the pediatric population.

However, a systematic review concluded that CIMT was significantly increased in 55 of the 67 studies, which included children with obesity, diabetes, hypertension, dyslipidemia, or chronic renal failure, despite assessment difficulties in children and the heterogeneity of the methods used. This finding highlights the potential utility of carotid ultrasonography to identify vascular changes, besides reinforcing that early vascular damage occurs in the pediatric population, and that vascular lesions might be amenable to early detection [25].

Investigators had found evidence that CIMT is stabilized or even decreased when cardiovascular risk factors such as hypercholesterolemia, glycemic control, or overweight are optimized, and this has also been shown in selected groups of pediatric patients [26].

Endothelial remodeling and consequent detection of carotid intima-media layer thickening in patients with diabetes are associated with longer disease duration, poor glycemic and lipid control, and high blood pressure [23, 27, 28].

We have shown that SBP percentile was associated with CIMT values, although DM1 adolescents evaluated in this study presented SBP and DBP within the normal ranges, according to reference standards. A strong association has been reported between increased CIMT in patients with DM1 and high SBP, stronger than that with LDL-C levels, family history of CVD, and diabetes duration [29, 30]. It had been suggested that among metabolic syndrome components, SBP is one of the most significant factors associated with increased CIMT [30, 31]. Increased CIMT has already been reported in normotensive children and adolescents with diabetes [32]. This finding supports the evidence that even normotensive individuals are at risk of arterial wall damage, depending on the degree of exposure to metabolic abnormalities, which could be detected by CIMT measurements, as was found in this group of DM1 patients. The association between the high-normal SBP values and increased intima-media thickness in normotensive adolescents might also support the existence of a continuous effect of blood pressure values on CIMT.

Other risk factors such as dyslipidemia are well established in the physiopathological mechanism of atherosclerosis and associated to increased CIMT in patients with DM1 [29, 33] as we were able to show. In the diabetes control and complications trial/epidemiology of diabetes interventions and Complications study, LDL-C levels, formation of immune complexes, oxidized LDL levels, and final products of enzymatic glycosylation of LDL were significantly associated with the progression of CIMT [34]. Increased CIMT was also reported in children with familial hypercholesterolemia and homocystinuria, metabolic diseases associated with premature atherosclerosis [35].

In the present study, an association between increased CIMT and borderline Total-C and LDL-C concentrations, in addition to the association with the highest values, draws attention to the difficulty of characterizing normality criteria for evaluation of lipid concentrations, mainly in children.

An unexpected finding was the lack of association between the HBA1c levels and CIMT. The study presented, however, a limitation to this evaluation. The glycemic control in the adolescents with diabetes was assessed only in the last year of monitoring, which probably could not have reflected the control of the disease throughout its duration. In prospective studies, when the long-term effects of intensive glycemic control on the progression of atherosclerosis were evaluated, the mean HBA1c level was associated with increased CIMT [34]. The benefits of adequate glycemic control have been widely established in individuals exposed to chronic hyperglycemia [36].

Moreover, in this study, disease duration was associated with increased CIMT, probably reflecting cumulative endothelial damage after exposure to chronic hyperglycemia and other risk factors since diagnosis. It was recently reported in patients with DM1 of long duration there was a 0.4% increase in IMT for each year of disease duration [37], as we were also able to demonstrate. Age and diabetes duration had an additive effect on the IMT of DM1 patients.

Another limitation is the absence of CIMT measures in young adolescents having diabetic vascular complications to ensure comparisons with the studied group. However these patients are rare.

The importance of assessing CIMT in a pediatric population is linked to the possibility of early intervention that leads to a better prognosis.

The reversibility of early atherosclerotic changes has already been reported in obese children who showed reduced CIMT after weight loss [26].

Guidelines have been established for screening for CVD risk factors in childhood, based on factors such as dyslipidemia, hypertension, and obesity. However, no consensus has been reached on the ideal age at which such screening should be conducted [38–40]. Although the reports on long-term outcome are still incipient, studies have suggested that therapeutic interventions in risk groups should be introduced as early as possible [41, 42] to reduce prolonged exposure to hemodynamic and metabolic insults that can lead to an irreversible pathological remodeling of the arterial wall.

Patients with diabetes present, since childhood, several factors that increase the risk of CVD, and it would be desirable that measures to identify, modify, and prevent the onset of cardiovascular events are taken early. The challenge lies in the absence of established parameters of normality for the evaluation of CIMT in this special age range, and also in the lack of a clear definition of the factors that influence its evolution, therefore prospective studies are needed.

It has been suggested that CIMT evaluation should be used at least as a tool for monitoring damage to target organs and therapeutic response in children and adolescents with high cardiovascular risk until the methodology for CIMT measurements is clearly defined [30]. Data presented here, also point in the same direction and reinforce the need for monitoring and maintaining a strict metabolic control in DM1 adolescents.

## Conclusion

Increased CIMT was observed in young Brazilian diabetic adolescents, and was associated with cardiovascular risk factors. We concluded that special attention should be given to early identification and monitoring of cardiovascular risk in young DM1 patients. CIMT assessment might be a useful instrument as CIMT has been considered an early sign of subclinical atherosclerosis in type 1 diabetes mellitus. Further longitudinal studies including larger number of patients are needed to verify these results.

## Abbreviations

BMI: body mass index
CCA: common carotid arteries
CIMT: carotid intima media thickness
CVD: cardiovascular diseases
DBP: diastolic blood pressure
DM: Diabetes Mellitus
HBA1c: glycated haemoglobin
HDL-C: high-density lipoprotein cholesterol
IMT: intima media thickness
LDL-C: low-density lipoprotein cholesterol
LCIMT: left carotid intima media thickness
RCIMT: right carotid intima media thickness
SBP: systolic blood pressure
SD: standard deviation
DM1: type 1 Diabetes Mellitus
WHO: World Health Organization
